# Prevalence of smoking cigarettes and beliefs regarding smoking habits among medical students: a cross-sectional study in Sudan

**DOI:** 10.3389/fpubh.2023.1193475

**Published:** 2023-06-20

**Authors:** Ahmed Abdalla Jarelnape, Waled Ahmed, Suaad Omer, Aida Fadlala, Zeinab Ali, Mohamed Hassan, Ragaa Ahmed, Manal Hakami, Mujtaba Ali, Khalid Mohammed, Elwaleed Sagiron, Yahya Hussein Abdalla, Abdalla Osman, Eltayeb Abdelazeem, Hamza Balola

**Affiliations:** ^1^Department of Nursing, Al Baha University, Al Bahah, Saudi Arabia; ^2^Faculty of Medicine, Al Baha University, Al Bahah, Saudi Arabia; ^3^Department of Community and Mental Health, Najran University, Najran, Saudi Arabia; ^4^Department of Nursing, University of Bisha, Bisha, Saudi Arabia; ^5^Department of Community, Batterjee Medical College, Khamis Mushait, Saudi Arabia

**Keywords:** smoking, prevalence, beliefs, medical students, Sudan

## Abstract

**Background:**

Smoking is a common problem in university students worldwide. Smoking is one of the most dangerous social phenomena and has a significant impact on public health. This study investigated the beliefs and attitudes of medical students toward smoking in Sudan.

**Methods:**

A cross-sectional study was conducted among medical students at Al Neelain University, Sudan, from March to June 2022 using a web-based questionnaire. The questionnaire consisted of eight items on demographic characteristics and 13 on the beliefs and attitudes toward smoking. Other data included smoking status, smoking habits, the number of cigarettes smoked per day, and smoking duration. Data analysis was performed descriptively, and chi-square test and logistic regression were conducted using SPSS version 24. Statistical significance was set at 0.05.

**Results:**

A total of 336 students participated in this study, and the smoking prevalence was 48.8% (41.1% in men and 7.7% in women). In total, 76.8% reported smoking daily at a rate of 5–10 cigarettes per day. In terms of students’ beliefs about smoking, 86.8% disagreed with selling cigarettes at the university. Of the respondents, 68.4% did not approve smoking on campus. There was a relationship between smoking habits and the age group of 22–25 years, which was the highest smoking category among students (*p*-value = 0.01).

**Conclusion:**

The prevalence of cigarette smoking among medical students is disturbing, particularly as they are future doctors. There is a need to include plans to reduce smoking among students that can be incorporated into courses and special programs.

## 1. Introduction

Smoking is a major risk factor for cardiovascular disease, respiratory disease, and stroke and is a major cause of death associated with these diseases (1). A study by Jha showed that smoking is considered the main factor leading death for patients with cardiovascular diseases (2).

Smoking is considered one of the greatest risks to public health in Africa. Because of its prevalence in the community, many African countries have appealed to the World Health Organization (WHO) to combat smoking (3). Previous studies have shown that smoking in Sudan affects 21% of those aged 18–30 years (4, 5). Smoking cigarettes was uncommon in Sudan at the beginning of the 19th century; however, at the beginning of the 20th century, it began to spread among young people (6). The most serious complications of smoking appear with age, and studies have shown that smoking in the prime of life leads to a higher likelihood of respiratory and cardiovascular diseases (7). In addition, smoking causes psychological, physical, social, and behavioral changes (8). Smoking has a wide effect on society, as nonsmokers are affected by exposure to smoke, which is called passive smoking (9). Smoking damages all body systems and may cause cancer (10). Moreover, smoking directly affects the standards of health, comfort, and happiness of individuals and groups (8).

Medical students should be role models in health, educate their peers about the effects of smoking and its impact on health, and represent the top of the pyramid regarding their impact on the beliefs and attitudes of others (11). Smoking among university students is usually due to imitation and influence of fellow smokers or accompanying groups. Students’ surrounding environment plays a key role in developing habits such as smoking (12). The prevalence of smoking among students may lead to weaknesses in their academic performance. A study in Sudan compared smoking and non-smoking students and found that students who smoked had lower academic achievement (13). According to a report by Dai on the prevalence and mortality related to smoking, the prevalence rate for smokers was 33% (14).

Among the studies described above regarding smoking among students, we found a scarcity of studies dealing with smoking among university students. Therefore, this study may stimulate researchers on the subject of smoking among students to come up with recommendations and guidelines that support the reduction in smoking habits.

Therefore, this study was conducted to investigate the beliefs and attitudes toward smoking among medical students in Sudan.

## 2. Materials and methods

### 2.1. Study design

This cross-sectional study was conducted among medical students from the Faculty of Medicine, Al Neelain University, Sudan.

### 2.2. Study setting and period

This study was conducted at the Faculty of Medicine, Al Neelain University, Sudan, from March to April 2022. There are 65 medical colleges in Sudan scattered between the capital and regions, 27 of which are governmental and 38 are private. The number of years of study in human medicine in Sudanese universities varies. The study period was 5 years in approximately 70% of universities and 6 years in the rest of the universities, with the addition of 12 months for clinical training. This period was referred to as the internship year and the medical profession only after the successful completion of that period.

### 2.3. Sampling and sample size

A simple random sampling method was used to enroll 435 students, 336 of whom completed the survey. The study sample included students of all academic levels and both sexes.

### 2.4. Data collection technique

Data were collected using an electronic questionnaire distributed to the students via email, which explained the study’s objectives. The participants were assured of the confidentiality of the collected data, including personal information. Responses were collected in an Excel spreadsheet and converted into SPSS for analysis.

### 2.5. Data collection tool

The questionnaire was divided into two sections. The first was for demographic information and characteristics of the participants, consisting of eight items. The second part consisted of 13 items that measured beliefs toward smoking among students. The questionnaire for this study was developed and modified by the authors of a previous study (15, 16) to suit the nature of the study population.

### 2.6. Pilot study

A pilot study was conducted with 15 students for opinions concerning the questionnaire for flexibility, time control, and their understanding of the vocabulary in the questionnaires. A pilot study sample was excluded. The results of the pilot test demonstrated the validity and reliability of the tool. Cronbach’s alpha was 0.77, indicating validity, which was shared with the study participants.

### 2.7. Statistical analysis

Data were analyzed using SPSS version 24 and were coded into variables. Frequencies and percentages were used to analyze the collected data. Statistical significance was set at *p* < 0.05.

### 2.8. Ethical considerations

Ethical approval was obtained from the ethics committee of Al Neelain University (Ethics approval code: ALN/20-2022). The participants received a cover letter explaining the purpose and outcome of the study and assuring them that their participation was voluntary. Informed consent was obtained from all the participants.

## 3. Results

### 3.1. Demographic characteristics of participants

The questionnaire was completed by 336 participants; 62.8% were men, and 37.2% were women. Most participants (70.5%) were aged 22–25 years. Results showed that the prevalence of smoking reached 48.8%, of whom 76.8% smoked daily, and their smoking rate per day ranged between 5 and 10 cigarettes; 73.8% of whom had smoked for 1–2 years. Results also showed that only 74.1% lived with their family (see Table 1).

**Table 1:** Demographic characteristics of participants.

		Frequency (*n*)	Percentage (%)
Sex	Male	211	62.8
	Female	125	37.2
Age categories (years)	18–22 years	55	16.4
	22–25 years	240	71.4
	More than 25 years	41	12.2
Academic years	First year	75	22.3
	Second year	66	19.6
	Third year	77	22.9
	Fourth year	63	18.7
	Fifth year	55	16.4
Student’s residence	With family	87	25.9
	Without family	249	74.1
Smoking status	Smoker	164	48.8
	Nonsmoker	172	51.2
Frequency of smoking habits	Every day	126	76.8
	Day after day	38	23.2
Number of cigarettes smoked per day	Less than 5 cigarettes	15	9.1
	5–10 cigarettes	126	76.8
	More than 10	23	14.0
Smoking duration	Less than 1 year	31	18.9
	1–2 years	121	73.8
	More than 2 years	12	7.3

### 3.2. Beliefs of students regarding smoking

Table 2 shows students’ beliefs regarding smoking habits. The findings of this study showed that 94.7% of the respondents disagreed with smoking habits. In total, 86.8% disagreed with the sale of cigarettes on university campuses. In total, 82.1% did not agree with smoking in closed places. Of the respondents, 68.4% did not approve of smoking on campus, whereas 92.8% believed that smoking affected their health.

**Table 2:** Beliefs of students regarding smoking.

	Strongly agree	Agree	Neutral	Disagree	Strongly disagree
	*n* %	*n* %	*n* %	*n* %	*n* %
Do you support smoking habits?	9(2.7)	4(1.2)	5(1.5)	16(4.8)	302(89.9)
Do you agree with the selling and marketing of cigarettes within university campuses?	19(5.6)	13(3.9)	12(3.6)	21(6.2)	271(80.6)
Do you agree with smoking in closed places?	17(5.1)	12(3.6)	31(9.2)	240(71.4)	36(10.7)
Do you believe smoking effects on health?	291(86.6)	21(6.2)	13(3.9)	4(1.2)	7(2.1)
Do you agree with smoking habits inside campuses?	68(20.2)	17(5.1)	21(6.2)	202(60.1)	28(8.3)

### 3.3. Relationship between smoking habits and age

Results revealed a relationship between smoking habits and age. There was a statistically significant difference between the number of cigarettes smoked per day and age category (*p*-value = 0.01). In addition, there were statistically significant differences in age groups and duration of smoking (*p*-value = 0.02). Duration of smoking did not differ significantly with age, as shown in Table 3.

**Table 3:** Relationship between smoking habits and age.

Correlation test				
	Age categories (years)			*P*-value
	18–22 years	22–25 years	More than 25 years	
	*n* %	*n* %	*n* %	
Number of cigarettes smoked per day				0.001^*^
1–5 cigarettes	4(2.4)	6(3.6)	5(3.1)	
6–10 cigarettes	22(13.4)	77(46.9)	27(16.5)	
More than 10 cigarettes	5(3.1)	15(9.1)	3(1.8)	
When did you start smoking?				0.671
Less than a year ago	5(3.1)	17(10.4)	9(5.5)	
1–2 years	3(1.8)	6(3.6)	3(1.8)	
More than 2 years	23(14.0)	75(45.7)	23(14.0)	
What is the frequency of your smoking?				0.021^*^
Every day	19(11.6)	83(50.6)	24(14.6)	
Day after day	12(7.3)	15(9.1)	11(6.7)	

* Statistically significant.

### 3.4. Relationship between smoking habits and sex

Results revealed a relationship between smoking habits and sex. Statistical significance was set at *p* < 0.05, as shown in Table 4.

**Table 4:** Relationship between smoking habits and sex.

Correlation test			
	Sex		*P*-value
	Male	Female	
	*n* %	*n* %	
Number of cigarettes smoked per day			0.000^*^
1–5 cigarettes	10(6.1)	5(3.0)	
6–10 cigarettes	114(69.5)	12(7.3)	
More than 10 cigarettes	14(8.5)	9(5.5)	
When did you start smoking?			0.000^*^
Less than a year ago	18(11.0)	13(7.9)	
1–2 years	112(68.3)	9(5.5)	
More than 2 years	8(4.9)	4(2.4)	
What is the frequency of your smoking?			0.001^*^
Every day	111(67.7)	15(9.1)	
Day after day	27(16.5)	11(6.7)	

* Statistically significant correlation.

### 3.5. Relationship between smoking habits and academic year

Table 5 shows the relationship between smokers’ characteristics and academic year. Statistical significance was found between the academic year of students and smoking period (*p*-value = 0.01).

**Table 5:** Relationship between smoking habits and academic year.

Correlation test						
	Academic year					*P*-value
	First year	Second year	Third year	Fourth year	Fifth year	
	*n* %	*n* %	*n* %	*n* %	*n* %	
Number of cigarettes smoked each day						0.891
1–5 cigarettes	3(1.8)	3(1.8)	4(2.4)	3(1.8)	2(1.2)	
6–10 cigarettes	36(21.9)	34(20.7)	43(26.2)	7(4.3)	6(3.6)	
More than 10 cigarettes	8(4.9)	4(2.4)	4(2.4)	4(2.4)	3(1.8)	
When did you start smoking?						0.001^*^
Less than a year ago	8(4.9)	10(6.1)	9(5.5)	3(1.8)	1(0.6)	
1–2 years	37(22.6)	35(21.3)	34(20.7)	8(4.9)	7(4.3)	
More than 2 years	3(1.8)	2(1.2)	5(3.0)	0(0.0)	2(1.2)	
What is the frequency of your smoking?						0.001^*^
Every day	35(21.3)	36(21.9)	39(23.8)	9(5.5)	7(4.3)	
Day after day	12(7.3)	5(3.0)	12(7.3)	5(3.0)	4(2.4)	

* Statistically significant correlation.

### 3.6. Logistic regression analysis test for smoking students

A logistic regression test was conducted to examine the relationships between independent variables such as sex, age, academic year, frequency of smoking habits, number of cigarettes smoked per day, and smoking duration. Results showed a statistically significant *p*-value of less than 0.05 (Table 6).

**Table 6:** Logistic regression analysis test for students who smoked.

Variables		Odds ratio	95% CI	*P*-value
		Exp (B)	Upper–Lower	
Sex	Male	1.450	1.101–1.483	0.001^*^
	Female			
Age categories (years)	18–22 years	1.430	1.301–1.489	0.001^*^
	22–25 years			
	More than 25 years			
Frequency of smoking habits	Every day	0.898	0.688–1.244	0.572
	Day after day			
Number of cigarettes smoked per day	Less than 5			
	5–10 cigarettes	1.339	1.203–1.485	0.001^*^
	More than 10			
Smoking duration	Less than 1 year	1.361	1.205–1.486	0.001^*^
	1–2 years			
	More than 2 years			

* Statistically significant.

## 4. Discussion

Smoking directly affects health, and studies have shown that smoking poses a risk for many diseases, particularly cardiovascular and lung cancer (17). Half of the participants were smokers (48.8%). This finding is based on a study in Palestine by Bin Abdulrahman (18) who showed that smoking prevalence was 42% among students. However, this differs from other studies conducted by Ivana at the University of Banja Luka, Srpska, in which the prevalence of smoking among students was 75% (19). Another study was conducted at a university in Jordan, where 28% of the students were smokers (20). The results of the study showed that 41.1% of men smoked. This result is similar to that of a study conducted on smoking among students at Al-Ghad Colleges in Saudi Arabia, which showed that 42% of students smoked (21).

Regarding the age of students who smoked, the study results showed that the age groups (18–22 years) were smokers compared to the others (67.1%). This result is supported and consistent with the study by Ghenadenik, and the results indicate that smoking spreads in this age group (22).

The findings of this study showed that three-quarters of students who smoked (74%) did not live with their families, and students’ residence with their families during the study period played a significant role in the emergence of habits such as smoking. This is similar to a study of medical students in Riyadh, Saudi Arabia, which found that approximately half of the smokers did not live with their families (23).

The results of the study indicate that the number of cigarettes smoked was 5–10 per day by 76.8% of the participants, which was consistent with the results of Wamamili’s study conducted at a New Zealand university where students smoked approximately 10 cigarettes per day (24). Smoking was practiced daily by 76.8% of the students; these findings were based on a study conducted in New Zealand, where 80% of students smoked daily (24).

The study results indicated that most of the students (82.1%) did not agree with smoking in closed places. This result was similar to that of a study conducted in New Zealand, in which 86% of the participants did not smoke indoors (24).

Medical students are usually more aware of the effects of smoking and health risks than other students. Therefore, in this study, most participants (94%) did not support smoking. This result is similar to that of a study conducted in Yemen, in which 85% of the students disagreed with smoking habits (25).

Regarding the selling and marketing of cigarettes on university campuses, the results showed that 86.8% of the students strongly disagree with selling cigarettes on campuses. This result agreed with a study conducted in Turkey, which showed that most students (83%) did not approve of selling cigarettes in universities (26). Among the recommendations of this study was the establishment of a program and clinics within the university that support smoking cessation, which was consistent with a study conducted in Irbid City, indicating a scarcity of smoking cessation policies and measures (27).

The strength of this study lies in the scarcity of studies on this subject. In addition, it deals with an important and widespread issue among young people, which is the subject of smoking and their beliefs about it. Numerous studies have shown its serious health effects, and the strength of this study lies in identifying beliefs and attitudes toward smoking among students. This study may contribute to the recommendations for the development of regulations and rules to reduce smoking among students. Medical students represent the first line of defense against harmful habits, such as smoking. Therefore, the prevalence of smoking and smoking habits among medical students in Sudan are of great importance in planning to reduce smoking.

## 5. Conclusion

The prevalence of smoking among students was 48.8%. More than half of the participants smoked an average of 5–10 cigarettes per day. University students represent the core of the future, including medical students, who represent the first line of defense and provide guidance against negative habits, such as smoking. Therefore, restrictions and instructions regarding smoking within universities must be applied, which can be included in the course of the study and its programs. Evaluating the effectiveness of smoking cessation programs and other interventions aimed to reduce smoking among medical students. This can help identify effective strategies for reducing the prevalence of smoking and beliefs about smoking among this population. Universities should consider efforts to raise awareness of smoking cessation and start operating smoking cessation clinics.

## 6. Limitations

This study has limitations and strengths, including its cross-sectional design, small sample size, and the fact that the study is conducted at a single educational facility, the Faculty of Medicine at Al Neelain University in Khartoum, Sudan. Consequently, the findings cannot be applied to all medical students in Sudan, and selection bias limits the generalizability of the study findings. In light of the paucity of studies on smoking habits and attitudes toward it in developing countries, particularly in Africa, this study might add value to the literature.

## Data availability statement

The raw data supporting the conclusions of this article will be made available by the authors, without undue reservation.

## Ethics statement

An ethical approval letter has been obtained from the Ethics Committee of AL-Neelain University (ethics approval code: ALN/20-2022). Informed consent was obtained from all participants, the study’s objectives were fully described, and participants were allowed to withdraw at any time. The patients/participants provided their written informed consent to participate in this study.

## Author contributions

AJ and SO wrote the main manuscript. AF, ZA, ES, AO, EA, HB, MA, and KM prepared the tables and performed data collection and analysis. All authors contributed to the article and approved the submitted version.

## Conflict of interest

The authors declare that the research was conducted in the absence of any commercial or financial relationships that can be construed as potential conflicts of interest.

## Publisher’s note

All claims expressed in this article are solely those of the authors and do not necessarily represent those of their affiliated organizations, or those of the publisher, the editors and the reviewers. Any product that may be evaluated in this article, or claim that may be made by its manufacturer, is not guaranteed or endorsed by the publisher.
